# Glyphosate Exposure Induces Cytotoxicity, Mitochondrial Dysfunction and Activation of ERα and ERβ Estrogen Receptors in Human Prostate PNT1A Cells

**DOI:** 10.3390/ijms25137039

**Published:** 2024-06-27

**Authors:** Teresa Chianese, Giovanna Trinchese, Rebecca Leandri, Maria De Falco, Maria Pina Mollica, Rosaria Scudiero, Luigi Rosati

**Affiliations:** 1 Department of Biology, University Federico II, Via Cintia 21, 80126 Napoli, Italy; teresa.chianese2@unina.it (T.C.); giovanna.trinchese@unina.it (G.T.); r.leandri8@gmail.com (R.L.); madefalco@unina.it (M.D.F.); mariapina.mollica@unina.it (M.P.M.); rosaria.scudiero@unina.it (R.S.); 2 National Institute of Biostructures and Biosystems (INBB), Viale delle Medaglie d’Oro 305, 00136 Roma, Italy; 3 BAT Center—Interuniversity Center for Studies on Bioinspired Agro-Environmental Technology, University of Napoli Federico II, 80055 Portici, Italy; 4 CIRAM—Centro Interdipartimentale di Ricerca “Ambiente”, University Federico II, Via Mezzocannone 16, 80134 Napoli, Italy

**Keywords:** estrogen receptors, glyphosate, human prostate cells, apoptosis, mitochondria

## Abstract

Glyphosate, the active ingredient of several broad-spectrum herbicides, is widely used throughout the world, although many adverse effects are known. Among these, it has been recognized as an endocrine disruptor. This work aimed to test the effects and potential endocrine disrupting action of glyphosate on PNT1A human prostate cells, an immortalized non-tumor epithelial cell line, possessing both ERα and ERβ estrogen receptors. The results showed that glyphosate induces cytotoxicity, mitochondrial dysfunction, and rapid activation of ERα and ERβ via nuclear translocation. Molecular analysis indicated a possible involvement of apoptosis in glyphosate-induced cytotoxicology. The apoptotic process could be attributed to alterations in mitochondrial metabolism; therefore, the main parameters of mitochondrial functionality were investigated using the Seahorse analyzer. Impaired mitochondrial function was observed in glyphosate-treated cells, with reductions in ATP production, spare respiratory capacity, and proton leakage, along with increased efficiency of mitochondrial coupling. Finally, the results of immunofluorescence analysis demonstrated that glyphosate acts as an estrogen disruptor determining the nuclear translocation of both ERs. Nuclear translocation occurred independent of dose, faster than the specific hormone, and persisted throughout treatment. In conclusion, the results collected show that in non-tumor prostate cells glyphosate can cause cell death and acts as a xenoestrogen, activating estrogen receptors. The consequent alteration of hormonal functions can have negative effects on the reproductive health of exposed animals, compromising their fertility.

## 1. Introduction

Glyphosate [Gly, N-(phosphonomethyl) glycine] is widely used in agriculture, horticulture, forestry, and other fields, given the ever-increasing demand for food closely related to the population increase that we are experiencing [1]. Today, although many harmful effects of Gly are well known [2,3], it continues to be used in many countries due to its formidable herbicidal properties, generally in the form of its best-known commercial formulation, Roundup [4]. As a result, we find Gly and its metabolite aminomethylphosphonic acid (AMPA) [5] in surrounding ecosystems, causing contamination of soil, water, and crops [6]. Gly does not remain confined to ecosystems but reaches animals, including humans, through the food chain, posing serious risks to the health of populations [7,8,9].

Among various adverse effects, Gly has been recognized as a substance that acts as a potential endocrine disrupting chemical (EDC) by altering hormonal homeostasis, modifying the localization and expression of estrogen receptors ERα and ERβ [10,11,12,13,14], and also promoting their activation [15,16].

Endocrine disruption represents an environmental and public health problem [17]. Evidence demonstrates that animal exposure to EDCs is associated with adverse health outcomes, including reduced fertility due to impaired gametogenesis, reproductive organ defects in offspring, and changes in the onset of puberty; in humans, exposure to EDCs is also associated to endocrine-related neoplasm such as in prostate, uterine, and breast cancers [17,18,19,20].

The most predominant endogenous estrogen is 17β-estradiol (E_2_), a critical hormone involved in a wide variety of effects on various cells and organs [21]. The effects of E_2_ are exerted primarily by estrogen receptors alpha (ERα) and beta (ERβ), both members of the nuclear receptor superfamily of transcription factors [22,23,24]. These two steroid receptors in their inactive form are present in the cell cytoplasm; after E_2_ binding, ERs undergo a conformational change that causes dimerization and nuclear translocation [25]. Once in the nucleus, they can bind to the estrogen response elements (EREs) present in the promoter region of estrogen responsive genes, eliciting transcriptional responses; this type of activation is considered a direct genomic effect. However, E_2_ can also trigger rapid cytoplasmic signaling cascades that do not involve ERs binding directly to DNA, but rather a binding to transcription factors already bound to the promoter (non-genomic effects) [26,27].

Estrogens have significant direct and indirect effects on prostate gland development and homeostasis and are presumed to play a role in the etiology of prostatic diseases. Estrogens can interfere with the androgen-induced development, growth, and differentiation of the prostate [28]. The direct effects are mediated by ERα and ERβ; in the prostate, ERα is found primarily in prostatic stromal cells and ERβ in the prostatic epithelium [29]. It has been suggested that differential localization may explain the diverse effects of estrogens recorded in the prostate gland. In particular, it has been hypothesized that estrogen-induced stromal proliferation may be mediated by ERα, while ERβ may have an antiproliferative role, also promoting the differentiation of the prostatic epithelium [29]. Several studies have suggested the role of estrogens in normal and aberrant prostate cancer, alone or in synergy with androgens [30]. Epidemiological and experimental studies highlight a relationship between estrogens/xenoestrogens and the pathogenesis of prostate cancer [30,31].

It has been demonstrated that Gly and AMPA are able to inhibit cell growth in human cancer cells, including prostate cancer cells, but not in two immortalized human normal prostatic cells [32]. This effect is ascribed to the fact that Gly and AMPA are analogous to glycine, a nonessential amino acid that is reversibly converted from serine. Glycine has been shown to be consumed by rapidly proliferating cancer cell lines [33]. Gly and AMPA, therefore, could inhibit serine hydroxymethyltransferase, the enzyme responsible for serine–glycine conversion, thus decreasing the availability of intracellular glycine. On the other hand, the possible endocrine disrupting effects of Gly [10,11,12,13,14,15,16] could interfere with the normal behavior of prostate cells.

Given the different, and often contradictory, results on the effect of Gly on normal and tumor prostate cells, in this work, we decided to test the action of Gly on the PNT1A cell line, already the subject of numerous studies on the response of non-tumor pancreatic cells to EDCs [34,35], to add further insights into the effects of Gly as an EDC on the prostate and its involvement in male infertility. Our results show that in these cells, Gly induces cytotoxicity, increasing DNA fragmentation and the level of proapoptotic proteins. The analysis of mitochondrial metabolism demonstrates Gly-induced mitochondrial dysfunction, probably to be considered as a bioenergetic response to stress conditions. Finally, immunofluorescence and western blot show a rapid activation of estrogen receptors ERα and ERβ, allowing us to validate Gly as a typical endocrine disruptor.

## 2. Results

### 2.1. Effects of Glyphosate on Cell Viability and Toxicity

**Figure 1:**
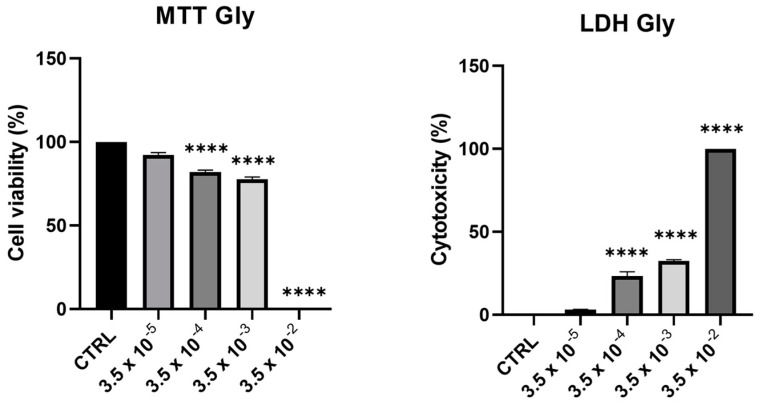
Assessment of cytotoxic effects of Gly on PNT1A cells. Cells were treated with increasing concentrations of Gly (from 3.5 × 10^−5^ M to 3.5 × 10^−2^ M) for 24 h. Cell viability was measured using MTT assay; cell cytotoxicity was measured using LDH assay. Values are reported as means ± SEM of three independent experiments, each in triplicate. CTRL: untreated (control) cells. Asterisks indicate statistically significant differences compared to untreated cells: **** *p* < 0.0001.

Cell viability and toxicity of PNT1A following 24-h treatment with Gly (starting from a concentration of 3.5 × 10^−5^ M up to 3.5 × 10^−2^ M) showed a dose-dependent decrease of viability, in parallel with an increase in cytotoxicity. At the maximum concentration tested (3.5 × 10^−2^ M), we recorded 100% of cell death (Figure 1).

From these data, we decided to use two concentrations of Gly for further investigations, namely 3.5 × 10^−4^ M (low dose, LD) and 3.5 × 10^−3^ M (high dose, HD).

### 2.2. DNA Fragmentation in Gly-Treated PNT1A Cells

**Figure 2:**
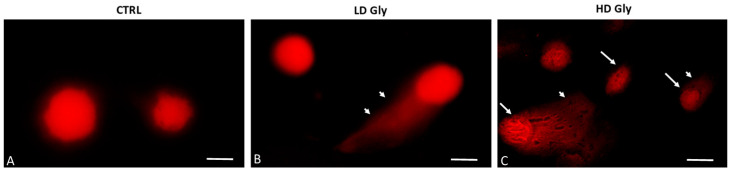
Representative images of comet assays in PNT1A cells exposed to different concentrations of Gly. (**A**) In the control samples (CTRL), cells with a of regular morphology of the nuclei are evident. (**B**) Comet formation is evident in PNT1A cells exposed to low dose (LD, 3.5 × 10^−4^ M) of Gly (arrowhead). (**C**) Tailed cells with highly fragmented DNA in the nucleus (arrows) are present in samples exposed to the high dose of Gly (HD, 3.5 × 10^−2^ M); arrowhead: comet formation. Scale bars correspond to 10 µm. Magnification 100×.

To establish whether Gly-induced cell death determined by the MTT assay involves nuclear DNA breakage and apoptosis, single-cell electrophoresis (comet assay) was performed on PNT1A cells exposed for 24 h to the two different concentrations of Gly previously selected. The comet assay is a versatile method for detecting nuclear DNA damage in individual eukaryotic cells; cells undergoing apoptosis exhibit a comet tail due to DNA fragmentation. In control samples, round-shaped cells with intact nuclei and no comets are evident (Figure 2A). Comets begin to be evident in prostate cells exposed to the low dose of Gly (Figure 2B); in samples exposed to the highest concentration of herbicide we recorded, in addition to the tailed cells, the presence of numerous cells that had a completely fragmented nucleus (Figure 2C).

### 2.3. Effects of Gly on the Protein Levels Involved in the Apoptotic Pathway

The triggering of the Gly-induced apoptotic pathway was confirmed by Western blot.

**Figure 3:**
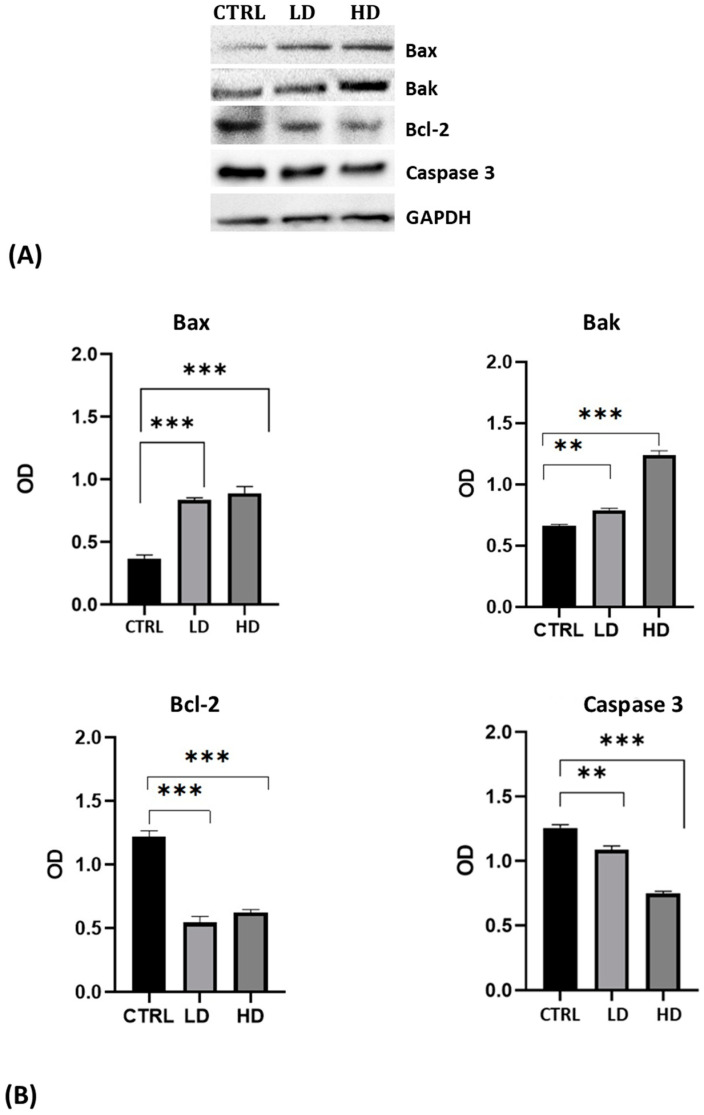
(**A**) Western blot analysis showing the levels of proteins involved in the apoptotic pathway extracted from whole lysate of PNT1A exposed for 24 h to low (LD, 3.5 × 10^−4^ M) and high doses (HD, 3.5 × 10^−2^ M) of Gly. CTRL: untreated cells. (**B**) Graphs showing the quantitative results for each protein. Protein levels were normalized against GAPDH levels. Values are means ± SEM of three independent experiments. Asterisks indicate statistically significant differences compared to control cells: ** *p* < 0.01; *** *p* < 0.001.

The analysis was performed using antibodies against the pro-apoptotic proteins Bax and Bak, the anti-apoptotic protein Bcl-2, and against the inactivated Caspase 3 protein. For all antibodies used, we recorded the presence of a single specific band of the expected molecular weight (Figure 3A). Results highlighted the increase in Bax and Bak levels and the decrease of Bcl-2 and of the uncleaved Caspase 3 in cells after 24 h of Gly treatment; the changes were more evident in cells treated with the high dose of Gly (Figure 3B).

### 2.4. Effects of Gly on Mitochondrial Metabolism

**Figure 4:**
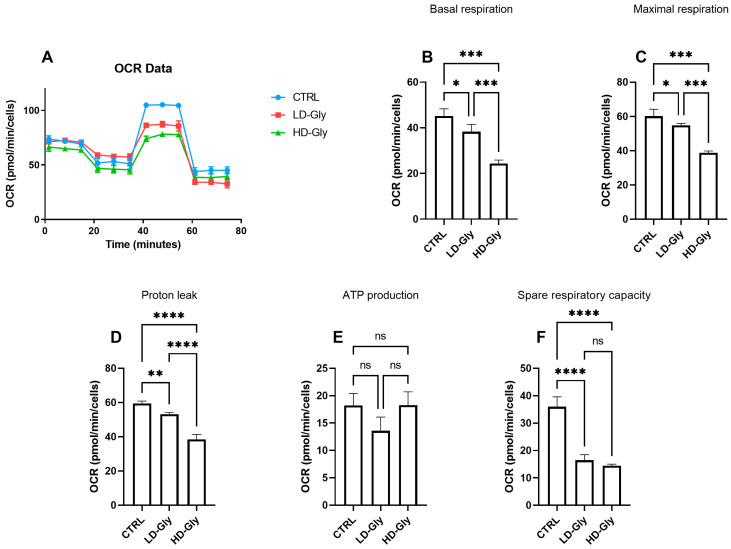
The representative graph of the Cell Mito Stress assay performed by the Seahorse XFp analyzer is reported (**A**). In the bar charts, each point in the OCR time courses is the average of three technical replicates. Basal respiration (**B**), maximal respiration (**C**), proton leak (**D**), ATP production (**E**), and spare respiratory capacity (**F**) are reported. The values are expressed as mean ± SD. Legend: CTRL, untreated cells; LD, low dose (3.5 × 10^−4^ M); HD, high dose (3.5 × 10^−2^ M). Asterisks indicate statistically significant differences respect to control cells: * *p* < 0.05, ** *p* < 0.01; *** *p* < 0.001, **** *p*< 0.0001; ns, not significant.

We determined the oxygen consumption rates (OCR) in PNT1A cells stimulated for 24 h with low or high doses of Gly (Figure 4A). We observed a significant reduction in basal (Figure 4B) and maximal (Figure 4C) respiratory rate in PNT1A cells stimulated with LD-Gly and, to a major extent, with HD-Gly, indicating a basic mitochondrial dysfunction that led to the impaired oxidative capacity of substrates. However, there was no significant variation in ATP production (Figure 4E) between the different groups.

It is presumed that in the LD and HD groups, the reduction in basal oxygen consumption rate was partly compensated by a significant reduction in mitochondrial proton leakage (Figure 4D), a mechanism that leads to an increase in coupling efficiency between substrate oxidation and oxidative phosphorylation, leading to the recovery of ATP production. These data are supported, at least in part, by the significant reduction in the spare respiratory capacity observed in LD- and HD-treated cells compared to control (Figure 4F), indicating the inability of cells to respond to a further increase in energy demand or under stress.

### 2.5. Effects of Gly on Cellular Localization of Estrogen Receptors in PNT1A Cells

To investigate whether Gly could activate the ER pathways, the cellular localization of both ERα and ERβ was observed by immunofluorescence after 30 min, 2 h, and 4 h of Gly treatment, at both low and high doses. To assay the estrogenic response of these cells, ERα and ERβ immunolocalization was observed after 30 min, 2 h, and 4 h of E_2_ treatment. Finally, analyses were performed treating the cells also in absence/presence of tamoxifen, a possible estrogen antagonist. In untreated PNT1A cells, ERα and ERβ were recorded exclusively in the cytoplasm.

#### 2.5.1. Cellular Localization of ERα in Gly-Treated PNT1A Cells

**Figure 5:**
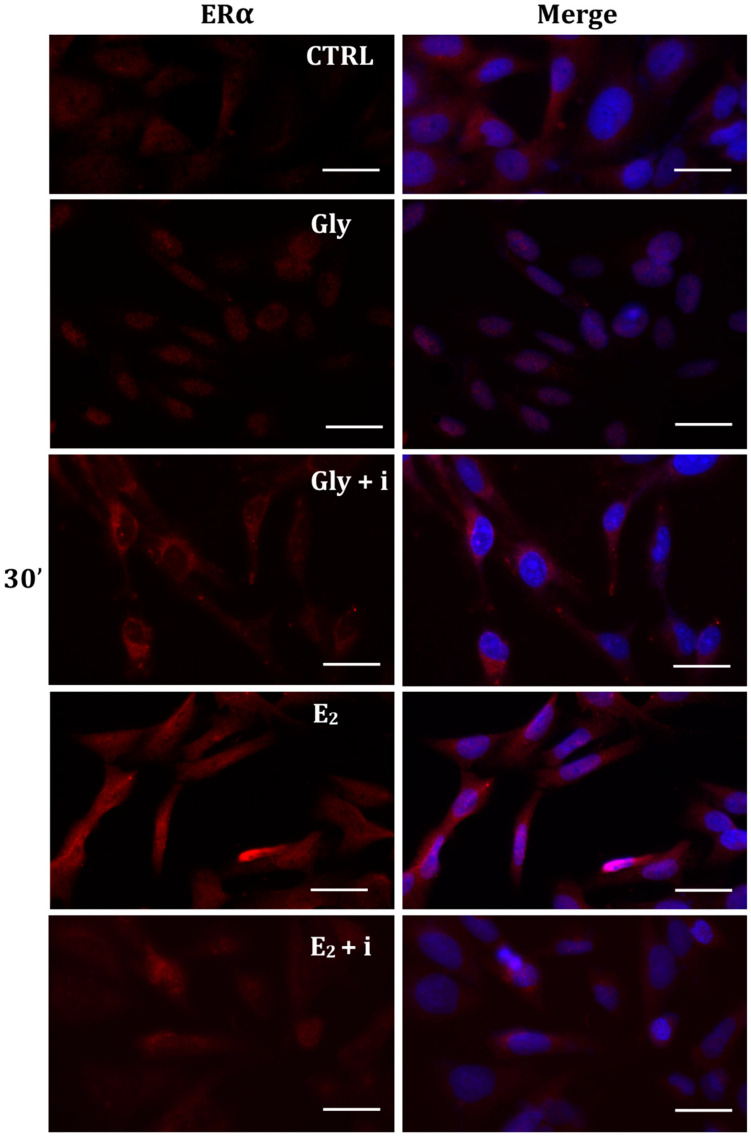
Immunolocalization of ERα after 30 min exposure to Gly 3.5 × 10^−4^ M (LD), E_2_ (10^−6^ M) alone, and in the presence of the inhibitor tamoxifen (E_2_ + i). CTRL: untreated PNT1A cells. The fluorescent signal appears red in color. The nuclei were stained with nuclear staining (Höechst, blue signal). Scale bars correspond to 20 µm. Magnification 40×.

**Figure 6:**
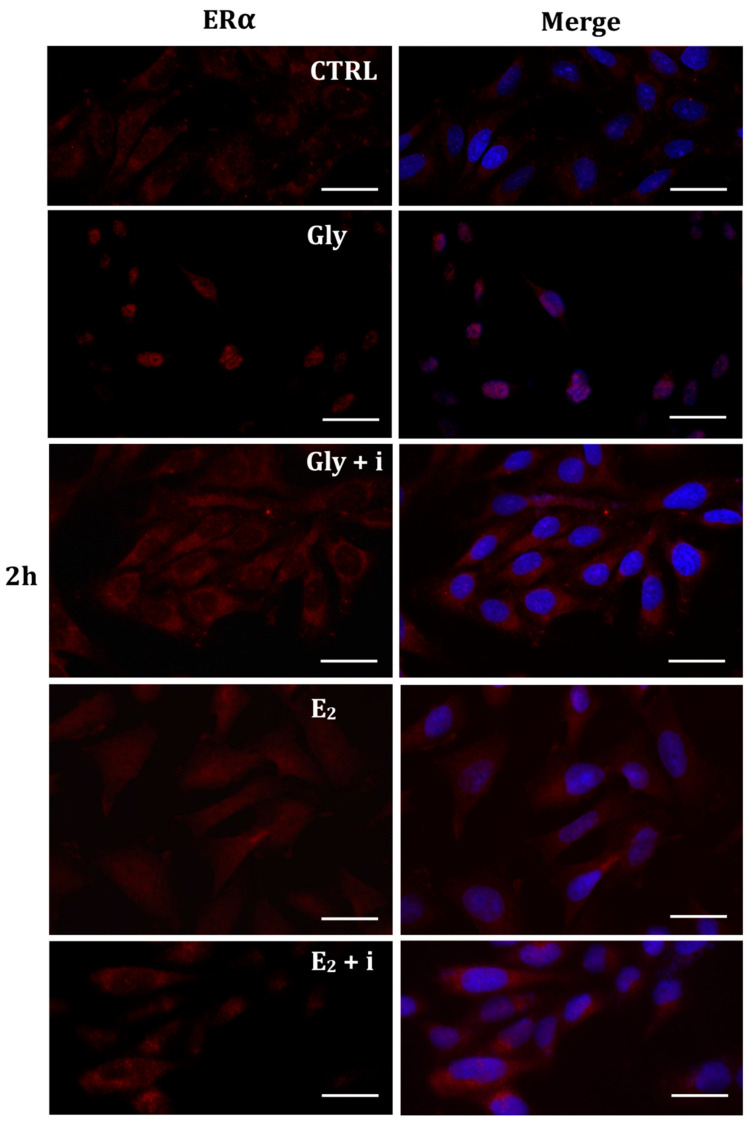
Immunolocalization of ERα after 2 h exposure to Gly 3.5 × 10^−4^ M (LD), E_2_ (10^−6^ M) alone, and in the presence of the inhibitor tamoxifen (E_2_ + i). CTRL: untreated PNT1A cells. The fluorescent signal appears red in color. The nuclei were stained with nuclear staining (Höechst, blue signal). Scale bars correspond to 20 µm. Magnification 40×.

**Figure 7:**
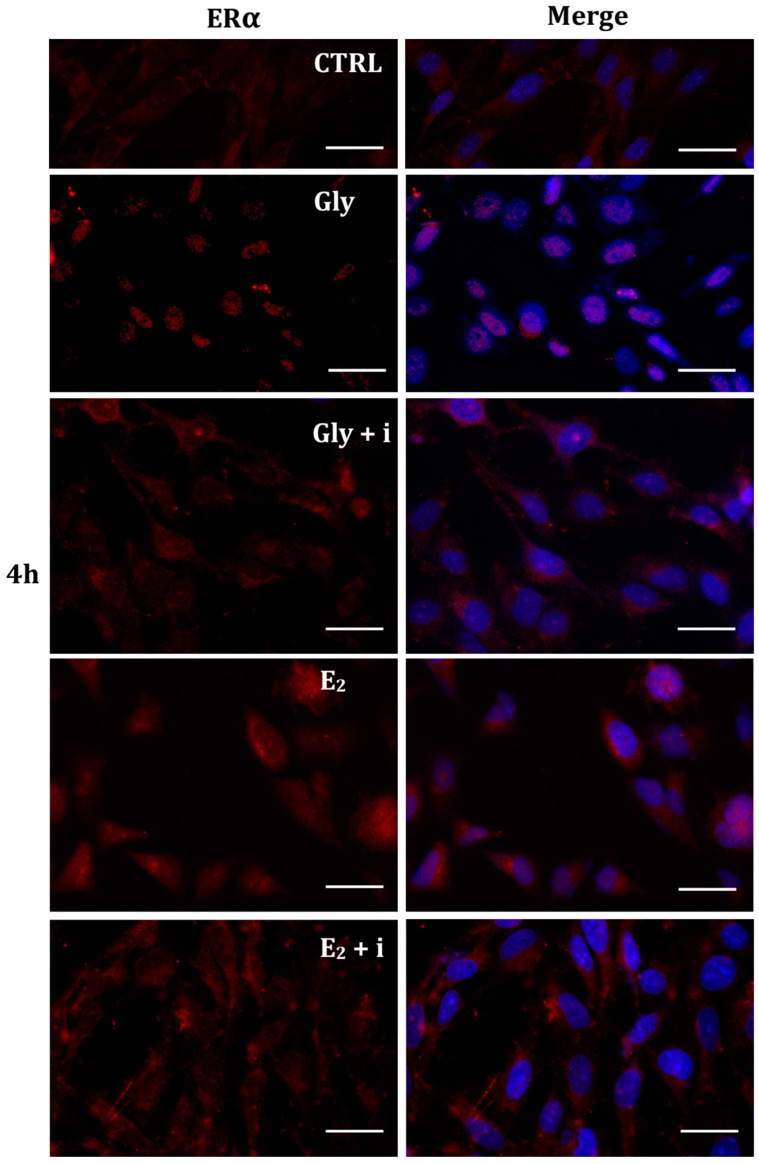
Immunolocalization of ERα after 4 h exposure to Gly 3.5 × 10^−4^ M (LD), E_2_ (10^−6^ M) alone, and in the presence of the inhibitor tamoxifen (E_2_ + i). CTRL: untreated PNT1A cells. The fluorescent signal appears red in color. The nuclei were stained with nuclear staining (Höechst, blue signal). Scale bars correspond to 20 µm. Magnification 40×.

The localization of ERα was determined by immunofluorescence analysis in control, E_2_-treated, and Gly-treated PNT1A cells, alone or in presence of tamoxifen after 30 min, 2 h, and 4 h. In all Gly-treated cells, regardless of the dose and duration of treatment, we recorded the ERα signal essentially in the nucleus. Figure 5, Figure 6 and Figure 7 show immunofluorescence images obtained after treatment with the low dose of Gly (3.5 × 10^−4^ M); images captured after high dose treatment are not shown, since they showed the same result as the low dose. Exposure of PNT1A cells to the endogenous estrogen E_2_ resulted in a more gradual activation of Erα; only after the most prolonged exposure (4 h), the receptor showed a predominantly nuclear localization (Figure 5, Figure 6 and Figure 7). Finally, the exposure of cells to tamoxifen prevented nuclear translocation: subsequent exposure of cells to Gly or E_2_ did not change the cytoplasmatic localization of the immunofluorescent signal, even after 4 h of treatment (Figure 5, Figure 6 and Figure 7). These results demonstrate that in PNT1A cells, as well as in prostate cancer cells [36], tamoxifen acts as an inhibitor rather than a modulator. Quantitative immunofluorescence intensity analysis is consistent and supports the results of ERα localization in PNT1A exposed to Gly alone and in the presence of tamoxifen, in both the cytoplasm and nucleus. In detail, after 30′ of Gly treatment, high levels of ERα immunofluorescence were recorded only in the nucleus. After 2 h of exposure, high levels of the receptor were detected, always in the nucleus, although ERα levels began to increase in the cytoplasm (Figure 11A). In fact, after 4 h of treatment a switch in the signal of the receptor profile was observed between the two cellular compartments, and ERα was found more in the cytoplasm than in the nucleus. As expected, in cells treated with Gly in the presence of tamoxifen, ERα immunofluorescence was assessed in the cytoplasm at all exposure times, as recorded in untreated cells (control) (Figure 11A).

#### 2.5.2. Cellular Localization of ERβ in Gly-Treated PNT1A Cells

**Figure 8:**
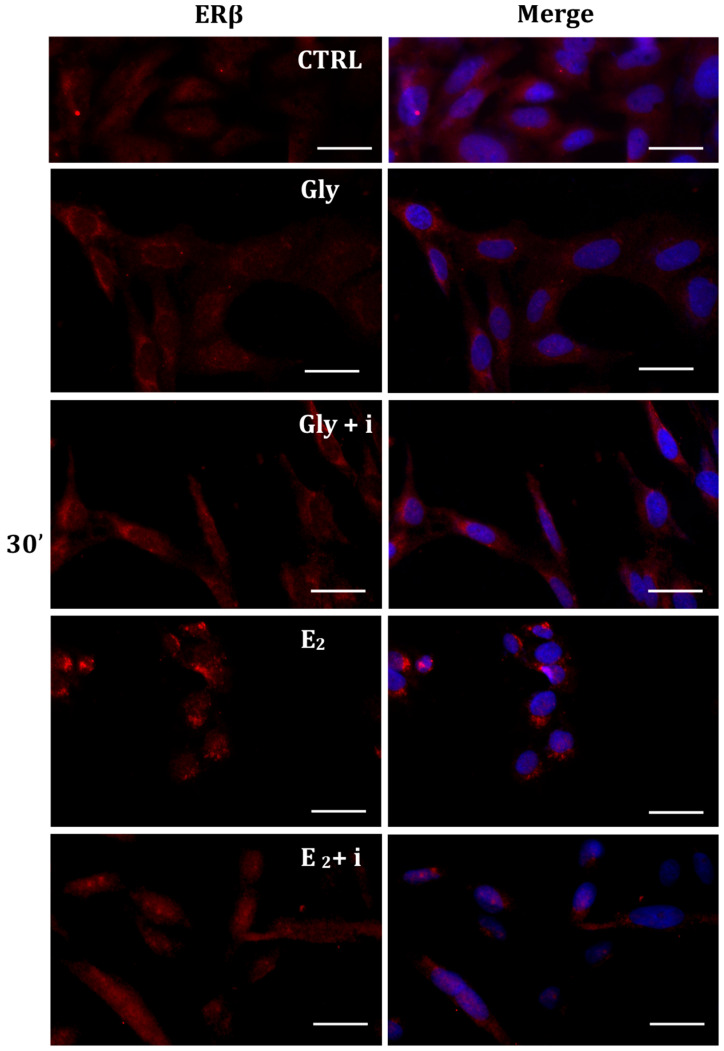
Immunolocalization of ERβ after 30 min exposure to Gly 3.5 × 10^−4^ M (LD), E_2_ (10^−6^ M) alone, and in the presence of the inhibitor tamoxifen (E_2_ + i). CTRL: untreated PNT1A cells. The fluorescent signal appears red in color. The nuclei were stained with nuclear staining (Höechst, blue signal). Scale bars correspond to 20 µm. Magnification 40×.

**Figure 9:**
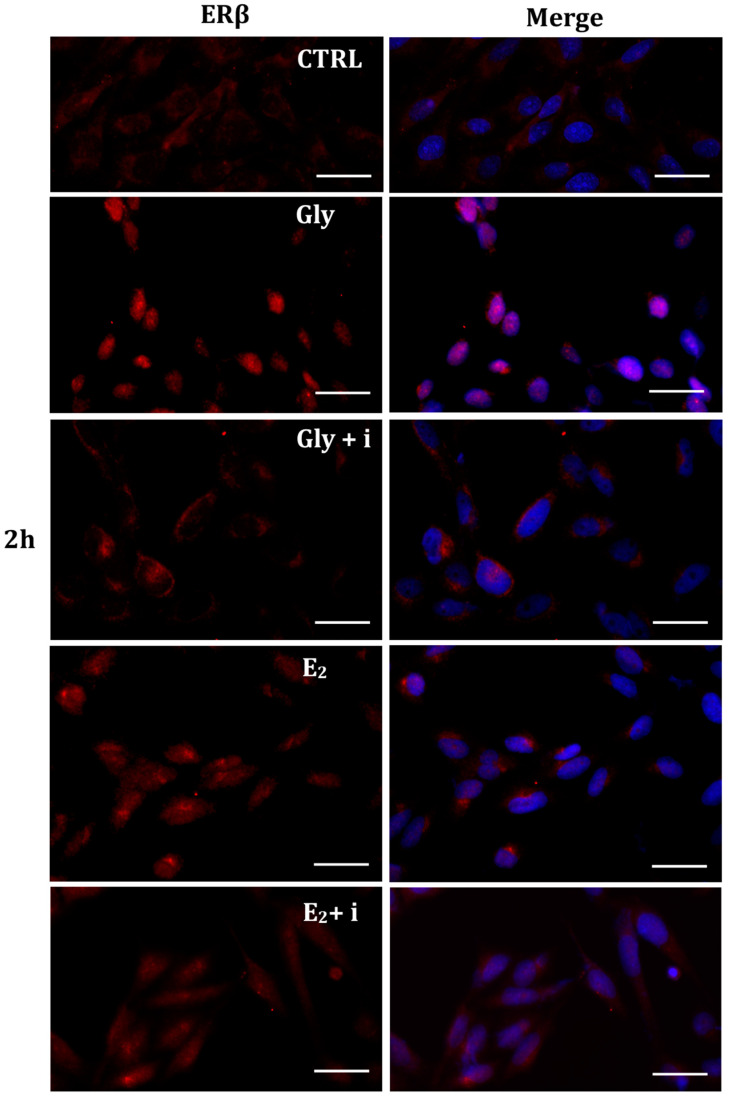
Immunolocalization of ERβ after 2 h exposure to Gly 3.5 × 10^−4^ M (LD), E_2_ (10^−6^ M) alone, and in the presence of the inhibitor tamoxifen (E_2_ + i). CTRL: untreated PNT1A cells. The fluorescent signal appears red in color. The nuclei were stained with nuclear staining (Höechst, blue signal). Scale bars correspond to 20 µm. Magnification 40×.

**Figure 10:**
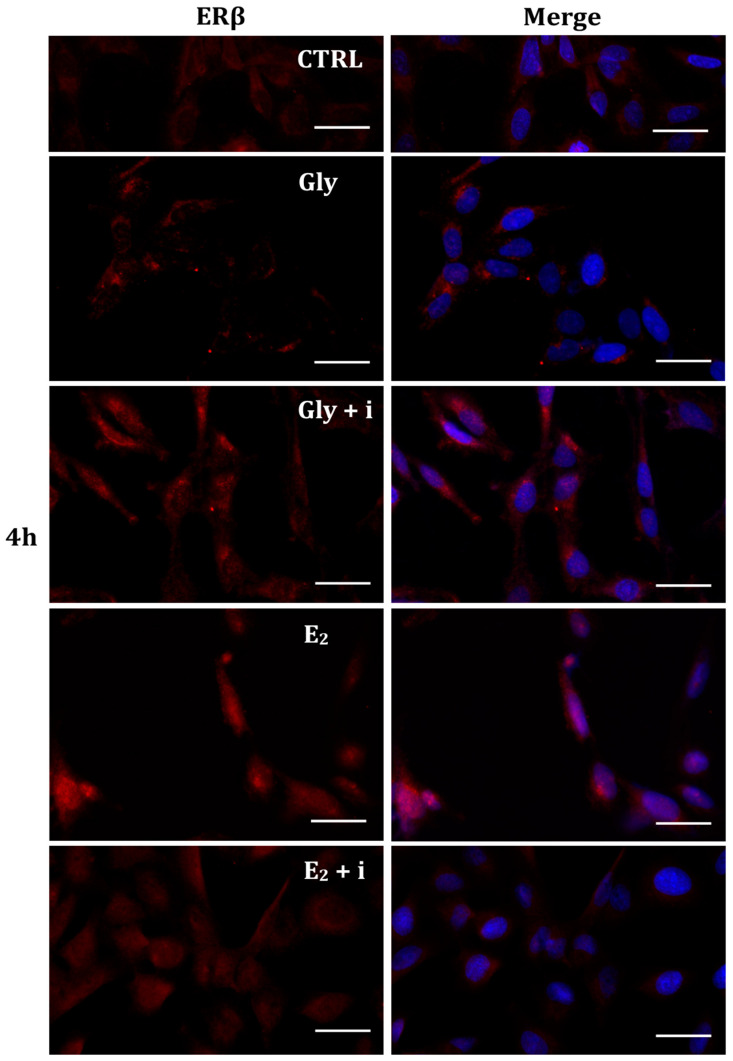
Immunolocalization of ERβ after 4 h exposure to Gly 3.5 × 10^−4^ M (LD), E_2_ (10^−6^ M) alone, and in the presence of the inhibitor tamoxifen (E_2_ + i). CTRL: untreated PNT1A cells. The fluorescent signal appears red in color. The nuclei were stained with nuclear staining (Höechst, blue signal). Scale bars correspond to 20 µm. Magnification 40×.

**Figure 11:**
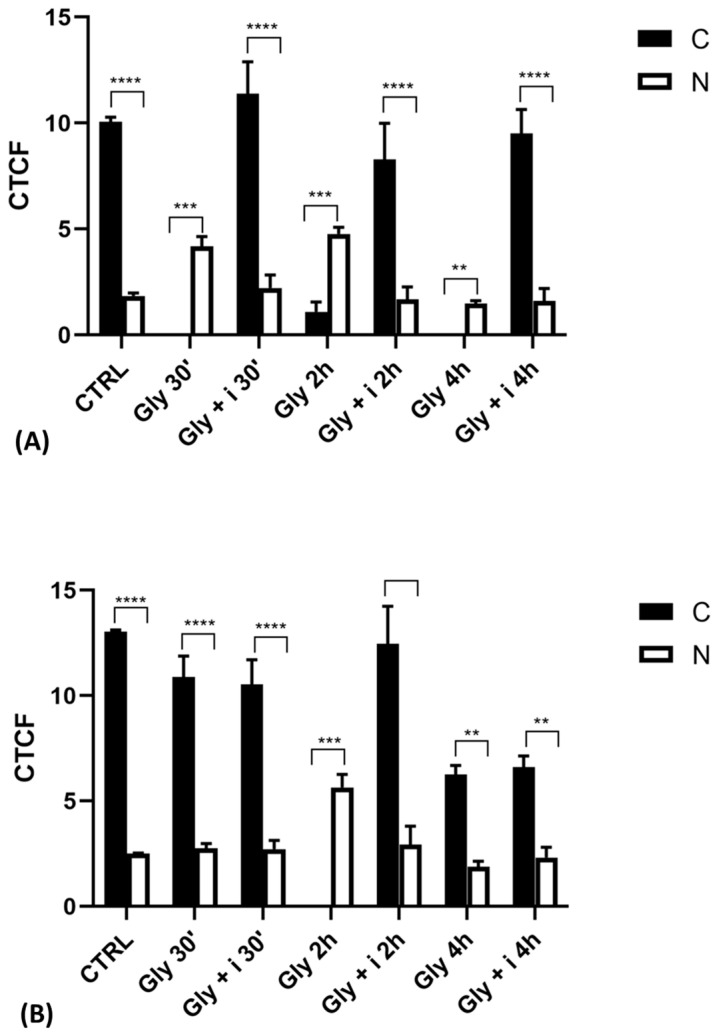
Graphs showing the results of immunofluorescence intensity analysis of ERs in the untreated PNT1A (CTRL) and in cells exposed to Gly (3.5 × 10^−4^ M) alone and in the presence of the inhibitor tamoxifen for different times. (**A**) ERα levels. (**B**) ERβ levels. Legend: C—cytoplasm; N—nucleus. Asterisks indicate statistically significant differences between receptor levels in the cytoplasm and nucleus for a specific exposure time: ** *p* < 0.01; *** *p* < 0.001, **** *p* < 0.0001.

Gly-induced ERβ localization showed slightly different behavior compared to ERα; however, again, the ERβ response was independent of Gly concentration. After 30 min of Gly treatment, no matter the dose, the fluorescent signal remained localized in the cytoplasm (Figure 8); after 2 h, ERβ translocated into the nucleus (Figure 9), and after 4 h, the receptor was colocalized in both the nucleus and cytoplasm, suggesting the cytoplasmic restoration of Erβ (Figure 10). As for ERα, the exposure of PNT1A cells to E_2_ determined a massive nuclear translocation of ERβ only after 4 h of treatment, while tamoxifen blocked its translocation, regardless of treatment (Figure 8, Figure 9 and Figure 10). As described for ERα, quantitative immunofluorescence intensity analysis supports the findings of ERβ localization in PNT1A exposed to Gly alone and in the presence of tamoxifen in both the cytoplasm and nucleus (Figure 11B). In cells treated with Gly for 30′, the ERβ signal was predominantly cytoplasmic, as in untreated cells. After 2 h of exposure, however, ERβ signal was almost exclusively in the nucleus (Figure 11B). The signal returned to being quantitatively greater in the cytoplasm after 4 h of treatment. In the presence of tamoxifen, the immunofluorescence intensity was always in the cytoplasm at all exposure times, as in control cells (Figure 11B).

### 2.6. Cellular Levels of ERα and ERβ in Gly-Treated PNT1A Cells

**Figure 12:**
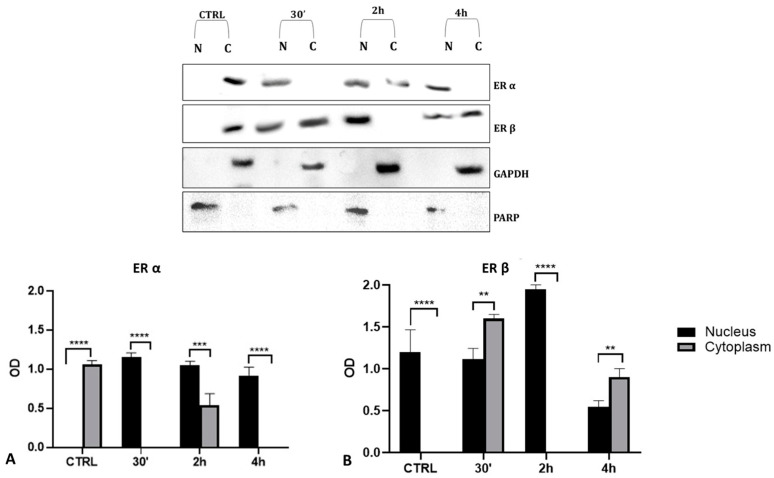
Western blot analysis of ERs levels in cytosolic and nuclear protein extracts of PNT1A cells. Representative Western blots of nuclear (N) and cytosolic (C) homogenates from control cells (CTRL) and cells treated for 30′, 2 h, and 4 h with 3.5 × 10^−4^ M (LD) Gly, stained with anti-ERα, anti-ERβ, anti-GAPDH, and anti-PARP. (**A**,**B**) Graphs showing the quantitative results for ERα (**A**) and ERβ (**B**). Nuclear protein level was normalized to PARP levels; cytosolic protein level was normalized to GAPDH levels. Values are means ± SEM of three independent experiments. Asterisks indicate statistically significant differences compared to control cells: ** *p* < 0.01; *** *p* < 0.001, **** *p* < 0.0001.

To validate the data observed with the immunolocalization, we performed a Western blot analysis on subcellular (cytosolic and nuclear) fractionated protein extracts from PNT1A cells exposed for 30′, 2 h, and 4 h to the low dose of herbicide. Analyses confirmed Gly-driven cytoplasmatic to nuclear translocation of estrogen receptors (Figure 12). In untreated cells, the two estrogen receptors were present only in the cytosolic fraction, for the entire time interval considered. On the contrary, in Gly-treated cells, we recorded a considerable increase in both ERα and ERβ signals in the nuclear fraction, starting already after 30′. The ERα signal was predominantly in the nuclear extracts at 30′ and 4 h of treatment (Figure 12A), while the ERβ signal was predominantly in the nuclear fraction after 2 h (Figure 12B).

## 3. Discussion

Thanks to recent scientific and technological discoveries, intensive agriculture characterized using pesticides and new equipment to increase food production has increasingly developed in recent decades. Intensive agriculture is aimed at satisfying a large food demand, due to the continuous increase in the world population [37]. To ensure greater food production, herbicides and pesticides are widely used to eliminate weeds, pathogenic organisms, and parasites from crops. At the same time, however, due to their diffusion, stability, toxicity, and bioaccumulation, they are among the most toxic substances that contaminate the environment today [38], consequently causing greater concern for human health, including reproduction [39,40].

For this very reason, this study focused its attention on Gly, which today is the most used herbicide in the world, to evaluate its effect on the reproductive capacity of non-target organisms. Many studies have highlighted glyphosate ability to act as a potential endocrine disrupting chemical by altering normal hormonal functions and mimicking the effects of E_2_ [41,42,43]. Recently, it was shown that Gly, although at high concentrations, promotes ERα phosphorylation and transcriptional activity in ERα-positive breast cancer cells [16].

Starting from these data, we decided to evaluate the ability of Gly to act as an EDC by activating the estrogen receptor pathway on PNT1A human prostate cells, a non-tumor cellular system consisting of both estrogen receptors ERα and ERβ [34,35].

The prostate gland plays a key role in male fertility. Its main function is to produce secretions (20–30% of total ejaculation) that provide the essential components of good quality seminal fluid. Androgens have a significant function in the development and differentiation of the prostate. Estrogens have also been shown to have direct effects on prostate gland development and adult homeostasis, but small variations in their levels or the presence of EDCs may play a role in the etiology of prostate disease [44].

The MTT and LDH assays highlighted Gly cytotoxicity. In particular, we observed a dose-dependent decrease in the percentage of cell viability and a corresponding increase in cytotoxicity. The assays allowed us to choose two different concentrations of herbicide which did not trigger cell death. These concentrations were used for the subsequent analyses, aimed at evaluating the triggering of Gly-mediated apoptosis, the impairment of mitochondrial metabolism induced by Gly, and the potential estrogen-like activity of Gly on these cells.

Single-cell electrophoresis demonstrated that the cytotoxic action of Gly against PNT1A cells resulted in DNA breakage, which, in turn, could be the first step in the activation of an apoptotic pathway. Western blot analyses showed that in Gly-treated cells the levels of the pro-apoptotic proteins Bax and Bak [45,46] were significantly increased, whereas the levels of the anti-apoptotic Bcl-2 [45,46] and the uncleaved caspase 3 [47] were decreased. Together, these data suggest the triggering in PNT1A cells of a Gly-mediated apoptotic process, as previously demonstrated in other cell lines [48,49,50], more responsive to the low dose than to the high dose. This result, which at first sight could be considered an anomalous behavior, can be explained by the observations that, in vivo, high levels of Gly are able to trigger inflammatory and fibrotic processes rather than regulated cell death such as apoptosis [9,13,14,51].

The triggering of apoptotic processes could be attributed to alterations in mitochondrial metabolism [52]; therefore, the main parameters of mitochondrial functionality were investigated using the Seahorse analyzer.

Mitochondrial metabolism must constantly adapt to cellular stress conditions to maintain bioenergetic levels adequate for cellular functions. Impaired mitochondrial function was observed in cells treated with both the low and high doses of Gly; however, this did not result in a significant change in ATP production levels, although a tendency towards reduction was observed in the group treated with the low dose of Gly, in line with what is a typical non-monotonic dose–response of endocrine disruptors.

It is presumable that the cells treated with Gly are distressed and therefore try to compensate for the Gly-induced alterations by working at high efficiency, as confirmed by the low values of mitochondrial proton leakage and the marked reduction in SRC, which indicates the inability of the PNT1A cells to bioenergetically adapt in response to stressful conditions. Furthermore, the high efficiency of mitochondrial coupling, dictated by low proton leakage, could lead to a pathological increase in ROS production resulting in oxidative stress which, in turn, would aggravate the cellular damage observed following Gly exposure. Further experiments should be performed to validate this hypothesis.

Finally, we verified the ability of Gly to act also in this non-tumor cell line as an EDC by investigating the activation of ERα and ERβ and comparing it with that determined by the endogenous hormone, 17β-estradiol. Estrogen-induced receptor activation is made evident by observing the translocation of the receptor from the cytoplasm to the nucleus [53].

The results of the immunofluorescence experiments that were allowed to follow the fluorescent signal linked to the antibody moving from the cytoplasm to the nucleus demonstrated that Gly in these cells acts as an estrogenic-like substance determining the nuclear translocation of both ERs. The nuclear translocation occurred independently of Gly dose and faster than the specific hormone. Indeed, the activation of both receptors by E_2_ was observed only after 4 h of treatment, when the fluorescence signal became predominantly nuclear, while the Gly-induced activation of ERα was already recorded after 30 min and of ERβ, after 2 h; in both cases, nuclear translocation of the receptors persisted throughout treatment. To verify whether this translocation was induced by a specific ligand–receptor interaction, we decided to use tamoxifen, that belongs to the family of selective estrogen receptor modulators (SERMs), molecules capable of selectively inhibiting or stimulating the estrogen-like action [54]. The concomitant presence of tamoxifen in the culture medium, both in the treatments with Gly and with E_2_, led to the lack of translocation of the receptors from the cytoplasm to the nucleus of PNT1A cells. These data were also confirmed by the evaluation of ER protein levels in nuclear and cytosolic extracts. In Gly-treated cells, the increase of both ERs in the nuclear subfraction with the concomitant decrease in the cytosol was evident; the kinetics of the nuclear translocation were also confirmed. This result, in addition to demonstrating that tamoxifen acts as an estrogenic inhibitor in this cell line, also shows that the translocation and activation of ERs induced by Gly are specific and comparable to that of E_2_, the natural substrate of these receptors.

Overall, the data described here could explain why the use of glyphosate is associated with the condition of infertility, given that, in addition to the direct damage to the gonads highlighted by many studies, both in vivo and in vitro [13,14,49,55,56], there are also structural and functional alterations of the accessory glands that are fundamental to produce functional seminal fluid, such as the prostate.

In conclusion, the results collected show that even in prostate cells, glyphosate directly acts as a xenoestrogen. Indeed, the results obtained from the immunofluorescence analysis clearly demonstrated that Gly can activate estrogen receptors, as does the endogenous hormone, showing faster receptor activation kinetics than the latter. This can be considered the first experimental evidence showing the interaction of Gly with both estrogen receptors modifying their activation. Moreover, glyphosate can induce cell death, mostly through apoptosis, probably induced by oxidative stress, as described for oocytes [49]. As an endocrine disrupting chemical, the resulting alteration of normal hormonal functions can have adverse effects on the health of non-target organisms, compromising, in particular, the reproductive process.

## 4. Materials and Methods

### 4.1. Chemicals

Glyphosate (Gly), 17β-estradiol (E_2_), and the selective estrogen modulator tamoxifen, a well-known estrogen antagonist for breast cancer [57], were purchased from Sigma-Aldrich (Sigma-Aldrich, St. Louis, MO, USA). Gly was dissolved in mQ water; E_2_ and tamoxifen were dissolved in pure DMSO (Invitrogen, Carlsbad, CA, USA). All the chemicals were diluted in a culture medium. In all the experiments with the estrogen antagonist, tamoxifen was added 1 h before the start of treatment at a higher concentration than Gly and E_2_. The final concentration of DMSO did not exceed 0.01% after scalar dilutions of E_2_ and tamoxifen in the medium.

### 4.2. Cell Culture

PNT1A cells (a non-tumoral human prostate cell line established by immortalization of adult prostate epithelial cells, ECACC 95012614) were cultured in Roswell Park Memorial Institute medium (RPMI, Sigma-Aldrich) with the addition of 10% fetal bovine serum (FBS, Sigma-Aldrich, St. Louis, MO, USA), 1% L-glutamine (Sigma-Aldrich, St. Louis, MO, USA), and 2% penicillin/streptomycin (Sigma-Aldrich, St. Louis, MO, USA) in an incubator at 37 °C and 5% CO_2_ at controlled humidity. Once confluent, cells were enzymatically detached with trypsin-ethylenediaminetetraacetic acid (EDTA) and cultured in new flasks. The medium was replaced every two days. Gly was added in the culture medium from a concentration of 3.5 × 10^−5^ M to 3.5 × 10^−2^ M, and E_2_ (a positive control) at a concentration of 10^−6^ M. In experiments involving the use of tamoxifen, it was added 1 h before the start of treatments at concentrations of 10^−5^ M for treatment with 10^−6^ M E_2_, and 10^−3^ M and 10^−2^ M for treatments with 3.5 × 10^−4^ M and 3.5 × 10^−3^ M Gly, respectively.

### 4.3. MTT Assay

The effects of Gly on PNT1A cell proliferation were evaluated using the 3-[4,5-dimethylthiazol-2-yl]-3,5-diphenyl-tetrazolium bromide (MTT) assay (Sigma-Aldrich, St. Louis, MO, USA). This method allows to measure cell viability, cell proliferation, and cytotoxicity. It is based on the capacity of mitochondrial oxidoreductases to reduce the tetrazolium dye MTT to an insoluble formazan precipitate, which has a purple color [58]. Cells were seeded in a 100 μL culture medium (5000 cells/well) in a 96-well plate. The next day, starvation was performed with an FBS-deprived medium (FBS 1%). Afterwards, the cells were incubated for 24 h with different compounds according to the experimental design. Gly was added at concentrations from 3.5 × 10^−2^ M to 3.5 × 10^−5^ M. At the end of the treatments, 10 μL of MTT solution (5 mg/mL) was added to each well. After 4 h of incubation at 37 °C, the culture medium was carefully removed and replaced with 100 μL of DMSO/isopropanol (1:1) to dissolve the formazan precipitates. The amount of formazan (directly proportional to the number of viable cells) was measured by recording the absorbance at 570 nm using a plate reading spectrophotometer (Synergy HTX Multi mode microplate reader, Agilent Technologies, Santa Clara, CA, USA). The % viability was calculated according to the following formula: (OD_490nm_ evaluated sample/OD_490nm_ negative control) = R; R × 100 = % cell viability.

### 4.4. LDH Assay

The toxicity of Gly on PNT1A cells was evaluated by the lactic acid dehydrogenase (LDH) assay (Dojindo Molecular Technologies, INC). The assay was carried out according to manufacturer’s protocol. Briefly, the cells were seeded in 100 μL of culture medium (5000 cells/well) in 96-well plates, incubated, starved, and treated as described for the MTT assay. The amount of LDH released from the cells in each well (directly proportional to the cytotoxicity of the treatment) was measured by recording changes in absorbance at 490 nm using a plate-reading spectrophotometer (Synergy HTX Multi mode microplate reader).

### 4.5. Comet Assay

The single cell gel electrophoresis (as known as the Comet assay) was performed as described by Simoniello et al. [59]. Briefly, a 50 µL cell suspension was mixed with 50 µL of 1% low melting point agarose and placed on slides previously coated with a layer of normal melting agarose. The slides were fixed at 4 °C for 10 min and then incubated for 2 h at 4 °C in a lysis solution (10 mM Tris, 0.1 M, EDTA, 2.5 M NaOH, 150 mM NaCl, 0.5% Triton-X 100, and pH 10) and then subjected to the electrophoretic run at 25 V and 300 mA for 30 min. Finally, the slides were washed in 1× PBS and then stained with ethidium bromide (5 µg/mL) for 5 min, after which they were mounted with a PBS/glycerol mix and observed using the Axioskop microscope (Carl Zeiss, Stuttgart, Germany) equipped with epifluorescence.

### 4.6. Seahorse XFp Analysis

Mitochondrial metabolism in PNT1A cells was assessed by the Seahorse XFp analyzer (Seahorse Biosciences, North Billerica, MA, USA) by using the Cell Mito Stress Test kit (Agilent, Santa Clara, CA, USA, cat# 103010-100). To test the effects of acute stimulation of 24 h with low or high doses of Gly (3.5 × 10^−4^ M and 3.5 × 10^−3^ M) on mitochondrial metabolism, cells were seeded in Seahorse mini-plates in complete RPMI medium (2 × 10^4^ cells/well). Before mito-stress analyses, the cells were centrifuged at room temperature at 1200 rpm for 10 min and the medium was replaced with a buffered base medium (Agilent Seahorse-103193, Agilent Technologies, Santa Clara, CA, USA) supplemented with 2 mM glutamine, 1 mM pyruvate, and 10 mM glucose at pH 7.4. The plates were centrifuged at 200× *g* for 5 min at room temperature and equilibrated at 37 °C in a CO_2_-free incubator for at least 1 h. Basal oxygen consumption rate (OCR) was determined in the presence of glutamine (2 mM) and pyruvate (1 mM). The proton leak was determined after inhibition of mitochondrial ATP production by 1 µM oligomycin, as an inhibitor of the F0-F1 ATPase. Furthermore, the measurement of the ATP production in the basal state was obtained from the decrease in respiration by inhibition of the ATP synthase with oligomycin. Afterward, the mitochondrial electron transport chain was stimulated maximally by the addition of the uncoupler FCCP (1 µM). Spare respiratory capacity (SRC) is the capacity of the cell to respond to an energetic demand and was calculated as the difference between the maximal respiration and basal respiration. The mitochondrial respiration was expressed as the oxygen consumption rate per minute normalized to the number of cells. In our experimental conditions, the same cell number/well was plated before the OCR measurements; the cell count was obtained by using the Burker chamber.

### 4.7. Immunofluorescence

The cellular location of ERs was determined by using indirect immunofluorescence. PNT1A cells were seeded in 8-well chamber slides (Sarstedt, Nürnbrecht, Germany) overnight at a density of 20,000 cells/well. After 24 h of serum-free (1% FBS) starvation, cells were treated with two different doses of Gly established based on MTT and LDH data (3.5 × 10^−4^ M and 3.5 × 10^−3^ M), and with E_2_ (10^−6^ M) for 30 min, 2 h, and 4 h, with or without tamoxifen (10^−5^ M, 10^−3^ M, and 10^−2^ M, respectively). At the end of each treatment, the medium was removed, the cells were washed with 200 μL PBS 1×, and fixed with 200 μL paraformaldehyde (4% in PBS 1×) for 15 min at room temperature. After 3 washes with cold PBS 1×, the cells were permeabilized with 0.1% Triton X-100 in 200 μL PBS 1× for 5 min to allow the ER antibodies to cross the cell membrane and reach the cytoplasm. After 3 quick washes with 200 μL of cold PBS 1×, cells were incubated with 1% bovine serum albumin (BSA) in 200 μL PBS 1× for 30 min at room temperature for blocking nonspecific sites. Afterwards, the chambers were incubated for 1 h at room temperature with the primary anti-ERα antibody (E-AB-66893 Elabscience rabbit polyclonal) and the anti-ERβ (SC-8974 Santacruz rabbit polyclonal), both diluted 1:100 in 1% BSA. Then, cells were incubated in the dark for 30 min at room temperature with the fluorescent secondary Goat anti-Rabbit IgG antibody DyLight 594 Conjugate (GtxRb-003-D594NHSX, ImmunoReagents, Raleigh, NC, USA, 1:200). After three washes with PBS 1×, cells were then treated for 2 min at room temperature with 0.5 μg/mL Höechst (Invitrogen) diluted 1:1000 in PBS 1× and then subjected again to three PBS washes. Finally, cells were mounted on slides with glycerol and PBS 1× (1:1). The negative control was obtained by omitting the primary antibody. Observations were performed with an Axioskop microscope (Carl Zeiss) equipped with epifluorescence, with the 40× objective, using two different filters: one for Höechst 33258 (excitation: 360 nm, blue emission: 452 nm) and another for AlexaFluor 594 (excitation: 594 nm, red emission: 614 nm). The images were acquired using an Axiocam MRc5 camera (Carl Zeiss) and the Axiovision 4.7 software (Carl Zeiss). Microscope fields with similar cell densities were chosen for the different experimental groups. The immunofluorescence analysis was performed in triplicate and different fields were chosen for data analysis.

### 4.8. Western Blotting

To determine protein levels, Western blotting was performed. For the total protein extractions, control cells and cells exposed for 24 h to 3.5 × 10^−4^ M and 3.5 × 10^−3^ M of Gly were harvested using a scraper and subjected to total protein extraction using the RIPA lysis buffer (Tris 50 mM, NaCl 150 mM, SDS 0.1%, Na Deoxycholate 0.5%, NaF 5 mM, NP40 1%, and EDTA 10 mM) enriched with a protease inhibitor cocktail (Sigma-Aldrich, St. Louis, MO, USA). For the subcellular fractionation (cytoplasm and nucleus), a protocol by Dimauro and coworkers was used [60]. Briefly, cells were homogenized with a homogenizer in an ice-cold STM lysis buffer (250 mM sucrose, 50 mM Tris-HCl pH 7.4, 5 mM MgCl_2_) and protease inhibitors. The resulting homogenate was centrifuged at 800× *g* for 15 min, thus obtaining a pellet containing nuclei and cellular debris, and a supernatant with cytosol and mitochondria. The pellet was resuspended in 8 mL of STM buffer, vortexed at maximum speed for 15 s and centrifuged at 500× *g* for 15 min. This step was repeated on the obtained pellet three times to increase the purity of the isolated nuclei, validated by microscopic inspection. The final nuclear pellet was resuspended in a NET buffer (20 mM HEPES pH 7.9, 1.5 mM MgCl_2_, 0.5 mM NaCl, 0.2 mM EDTA, 20% glycerol, 1% Triton-X-100, and protease inhibitors) and the nuclei lysed with 20 passages through an 18-gauge needle. The lysate was centrifuged at 9000× *g* for 30 min at 4 °C; the resulting supernatant, containing the nuclear proteins, was used for Western blot analysis. Cytosolic proteins and mitochondria were separated by centrifuging the starting supernatant at 11,000× *g* for 15 min. After centrifugation, the resulting pellet containing mitochondria was discarded; the supernatant, containing the cytosolic proteins, was used for Western blotting. The protein concentration in both the nuclear and cytosolic fractions was measured with the BCA protein assay (Thermo Fisher Scientific, Waltham, MA, USA), according to the manufacturer’s protocol. Then, 40 μg of protein extracts for each sample were boiled for 5 min in an SDS buffer (50 mM Tris-HCl (pH 6.8), 2 g 100 mL−1 SDS, 10% (*v*/*v*) glycerol, and 0.1 g 100 mL^−1^ Bromophenol blue), separated on 10% SDS-PAGE gels, and transferred to nitrocellulose membranes [61]. The membranes were blocked with two washes in 5% milk in TBS 1×-Tween, each at 40 min at room temperature. Afterwards, the membranes were incubated with primary antibodies diluted in TBS 1×-Tween overnight at 4 °C. The used polyclonal antibodies were rabbit anti-Bax (Elabscience, Houston, TX, USA, E-AB-66518, 1:500), rabbit anti-Bak (Elabscience, E-AB-70192, 1:500), rabbit anti-Bcl 2 (Elabscience, E-AB-60788, 1:500), rabbit uncleaved Caspase 3 (Elabscience, E-AB-66940, 1:500), rabbit anti-ERα (Elabscience, E-AB-66893, 1:300), rabbit anti-ERβ (Santacruz, SC-8974, 1:300), anti-GAPDH (Elabscience, E-AB-40516, 1:2000), and rabbit anti-PARP (Proteintech, Planegg-Martinsried, Germany, 13371-1-AP, 1:1000). Membranes were washed four times for 10 min in TBS 1×, 0.05% Tween-20, before a 1 h incubation with a secondary peroxidase-conjugated antibody (goat anti-rabbit IgG, Santa Cruz, Dallas, TX, USA, Sc-2005) diluted 1:2500 in TBS-T containing 2% BSA. After incubation, protein bands on the membrane were covered with a chemiluminescent HRP substrate (Amersham, Thermo Fisher Scientific, Milan, Italy) and visualized through a chemiluminescence scanner (ChemiDoc Image System, Bio-Rad Laboratories, Hercules, CA, USA). Western blot results were analyzed using ImageJ 1.54F to determine the optical density (OD) of the bands. The OD reading was normalized to GAPDH and PARP to account for variations in loading. When the same membrane was used to probe different primary antibodies, the membrane was stripped by incubation in 25 mM glycine, 1% SDS, and pH 2 at 50 °C for 30 min with shaking, followed by neutralization in TBS 1× and blocking with 5% milk in TBS 1×-Tween for 1 h at room temperature. The stripping yield was tested by evaluating the ECL signal after treatment with the stripping solution.

### 4.9. Statistical Analysis

The data were presented as means ± SD or SEM. Differences among all groups were compared by ANOVA, followed by the Bonferroni correction, and for Seahorse XFp analysis, the Newman–Keuls post hoc test to correct for multiple comparisons. Differences were considered statistically significant at *p* < 0.05. All analyses were performed using GraphPad Prism 5.0 (GraphPad Software, San Diego, CA, USA).

## Data Availability

Data supporting the findings of this study are available within the article.
